# Evidence for HPV DNA in the placenta of women who resorted to elective abortion

**DOI:** 10.1186/s12884-021-03937-9

**Published:** 2021-07-06

**Authors:** Maria Teresa Bruno, Salvatore Caruso, Francesca Bica, Giulia Arcidiacono, Sara Boemi

**Affiliations:** Department of General Surgery and Medical Surgery Specialties, Gynecological Clinic, University of Catania, Catania, Italy

**Keywords:** HPV infection, Placenta, Colposcopy, HPV DNA, Pregnancy, chorionic villi

## Abstract

**Background:**

It is believed that HPV infection can result in the death of placental trophoblasts and cause miscarriages or preterm birth. In clinical cases of placental villi positive for HPV DNA reported by other authors, contamination is suspected in the act of crossing the cervical canal. We analyzed placental samples of women who resorted to elective abortion obtained by hysterosuction of ovular material, bypassing any contact with the cervical canal and vagina.

**Methods:**

We studied the chorionic villi of the placenta of 64 women who resorted to voluntary termination of pregnancy, in the first trimester. To avoid contamination of the villi by the cervical canal, we analyzed placental samples obtained by hysterosuction of ovular material, bypassing any contact with the cervical canal and vagina. All samples of chorionic villi were manually selected from the aborted material and subjected to research for HPV DNA.

**Results:**

HPV DNA was detected in 10 out of 60 women (16.6%). The HPV DNA identified in the placenta belonged to genotypes 6, 16, 35, 53, and 90.

**Conclusion:**

The study shows that papillomavirus DNA can infect the placenta and that placenta HPV infection can occur as early as the first trimester of pregnancy.

**Supplementary Information:**

The online version contains supplementary material available at 10.1186/s12884-021-03937-9.

## Background

Human papillomavirus infection (HPV) is a sexually transmitted disease associated with cervical carcinogenesis whose prevalence of genotypes shows significant differences worldwide [1, 2]. In its single or multiple form [3] persistent high-risk human papilloma virus infection (hr HPV) is necessary [4] for progression of Cervical Intraepithelial Neoplasia (CIN) lesions. HPV infection is the most common sexually transmitted disease (STD) in the world, however, recent studies have shown that the infection can also be acquired by vertical transmission and through the placenta from mother to child [5, 6].

Recently, it was discovered that the HPV virus is capable of infecting trophoblast cells as well as squamous cells [7]. This discovery adding further support to the proposed association between HPV infection and miscarriage. In fact, trophoblast cells play a crucial role in the placentation process [8] and it is believed that HPV infection can result in the death of placental trophoblasts and cause miscarriages or preterm birth [9].

Evidence of HPV transmission through the placenta is not without controversy; in fact, HPV DNA was detected with a wide prevalence range of 4 to 75% in the placenta of women who miscarried and in 20–24% of women who underwent voluntary termination of pregnancy [10–12]. Eppel et al. [13] did not find HPV DNA in any of the 147 placental villi samples collected by transabdominal amniocentesis. In addition, in clinical cases of placental villi positive for HPV DNA reported by other studies, contamination is suspected in the act of crossing the vagina.

We investigated the presence of HPV in the placenta of women in the first trimester of pregnancy, who asked for termination of pregnancy (TOP), in order to verify the hypothesis that the placenta may be infected with HPV and that this manifests as early as the first trimester of pregnancy.

## Methods

From May 2019 to June 2020 we studied the chorionic villi of the placenta of 64 women who asked for TOP. The gestational age of women was between the 7th and 12th week. The decision on whether or not to participate in the study was at the patient’s discretion and the patient was not disadvantaged in any other way for refusing to participate in the study. Subjects were excluded if they had evidence of immunosuppression (HIV infection, transplantation, malignant tumor) or had been vaccinated against HPV. The women were invited to participate regardless of whether they had a history of HPV genital infection. The viral status (cervical HPV DNA) of the mother was not reported deliberately, first, because the aim of the research is to demonstrate the presence of HPV DNA in the placenta, second, because some data are incomplete and the risk is to reduce the number of women of the study group, already small. The prospective observational study was approved by the local institutional review committee (n. 154/2019/PO) and written informed consent was obtained from all study participants. Participants responded to a questionnaire with demographic and clinical data in order to characterize the study group (supplementary file).

Since other studies on vertical HPV transmission were often hampered by the possible contamination of the placenta with vaginal cells from an infected birth canal, other authors used the transabdominal pathway, whereas we analyzed placental samples obtained by hysterosuction of ovular material, bypassing any contact with the cervical canal and vagina.

Women were administred prostaglandins (geneprost 1 mg) by vaginal pessaries to dilate the cervix to such an extent that a cannula (Karman’s cannula) could be introduced in the uterus, which was connected to an aspirator to aspirate ovular material. In order to avoid contamination of the ovular material with HPV coming from the maternal vagina, we resorted to a particular method. We inserted a 10 mm Karman cannula into the already dilated cervix. This cannula acts as a shirt, through which a 6 mm Karman cannula reaches the uterine cavity and aspirates the ovular material, without contaminating it. Furthermore, the cervical canal lined with cylindrical cells, is hardly contaminated by papillomavirus as it has a particular tropism for the squamous epithelial cells that line the vagina and the uterine portio; this is even more so in pregnant women whose cervix is characterized by a physiological eversion of the endocervical mucosa on the uterine portio. Moreover, the cervix represents not only an anatomical barrier but also an immunological barrier against ascending infection through mucus production, inflammatory cytokines and antimicrobial peptides. All samples of chorionic villi were manually selected from the aborted material and subjected to research for HPV DNA.

### Samples and isolation of nucleic acid from chorionic villi

We have chosen to work on fresh, non-paraffinized placental tissue: in order to make the sample as homogeneous as possible. The placental tissue was processed in a sterile hood and, using a petri dish as a support, reduced to very small pieces using a scalpel from which 3 small sample sections from 3 different points were taken and placed into an Eppendorf type test tube. Following this, the treatment continued with cellular lysis by adding the lysis buffer (ATL buffer) and proteinase K with overnight incubation at 55 °C.

The day after the extraction continued using the commercial HIGH Pure PCR Template (ROCHE) kit which is based on the ability of DNA to bind to inert media or filters contained in columns; subsequently the nucleic acids bound to the filter were eluted through elution buffer and the eluate was stored at − 80 °C until the moment of use.

### DNA amplification

For the amplification of HPV DNA the Ampliquality kit HPV-TYPE EXPRESS v 3.0 (AB ANALITICA srl, Padova, Italy) was used. The kit is based on a rapid system for the identification of human Papillomavirus by single step PCR: in the preparation of the PCR session, in addition to the samples, a positive amplification and typing control (HPV 61 DNA) as well as a negative control (sterile distilled water) were also used; in addition, the kit can evaluate the suitability of the DNA extracted by the amplification of the TST gene (thiosulfate sulfurtransferase rhodanese).

The PCR mix was divided into a sufficient number of test tubes for the analysis of samples and controls, as follows:

HPV-TYPE EXPRESS MIX 20ul.

Extracted DNA 5ul.

The test tubes were then transferred to a thermocycler with the previously programmed amplification profile.

### DNA genotyping using reverse line blot

The subsequent genotyping of the amplified product was carried out by an allele-specific hybridization technique; it is a technique that includes a solid phase support in which specific probes (Reverse Line Blot) have been used and includes:

a- Denaturing: the marked amplified product is denatured by chemical or thermal denaturation (incubation at 95 °C);
b- Hybridization: the strip is incubated, under specific temperature conditions, agitation and phj with the solution containing the marked and denatured amplified product;
c- Washes: to remove the excess marked amplified product and then to define the “astringency” of the hybridization;
d- Detection: Displays the hybrid that was formed based on the type of marked probe (NBT/BCIP coloring solution).

The kit used for genotyping was Ampliquality HPV-TYPE EXPRESS, able to determine the presence of 40 types of Papillomavirus, in particular: 16, 18, 31, 33, 35, 39, 45, 51, 52, 56, 58, 59, 68, 73, 82, 26, 53, 66, 67, 6, 11, 40, 42, 43, 61, 69, 70, 44, 54, 55, 62, 64, 71, 72, 81, 83, 84, 87, 89, 90.

### Statistical analysis

The statistical analysis of the data was carried out with the software package SPSS 15.0 (SPSS Inc.; Chicago, IL, USA). Descriptive statistics were expressed by frequency, arithmetic mean and percentages.

## Results

### Four women were excluded from the study for insufficient chorionic villi obtined

Consequently, HPV DNA sequences were studied in 60 samples. HPV DNA was detected in 10 out of 60 women (16.6%). The HPV DNA identified in the placenta belonged to genotypes 6, 16, 35, 53, and 90. The villi of five placentas (50%) had high carcinogenic HPV (HPV 16, 53, and 35) and two (16.7%, *n* = 2/10) had two different types of HPV DNA. HPV 16 was the most frequent genotype in this study. Genotype 6 was the second most frequent (Table 1).

**Table 1:** Depict the age of the women, time of gestation and the type of the HPV (or HPVs) found in the chorionic villi of the 10 women tested

AGE	WEEK	HPV genotype
27	8	53
38	9	16
22	10	90
32	10	6, 56
25	9	35
26	11	6
27	9	67
22	10	16
36	11	66, 73
26	11	16

## Discussion

The aim of this study was to find evidence of HPV DNA in the placenta. Positive results from others anthors were affected by contamination of chorionic villi by a vaginal cells positive for papillomavirus [13]. To avoid contamination, authors such as Weyn [14] and Eppel used the transabdominal pathway to sample chorionic villi to be subjected to PCR. In our study, we analyzed the villi that come from the placentas of women who resorted to elective abortion and to avoid contamination of the villi by the vagina we used a procedure allowed us to aspirate ovular material through a cannula that was introduced through the already dilated cervical canal with the administration of local prostaglandins. Whereas the cervix represents not only an anatomical barrier but also an immunological barrier against ascending infection, this method allows the sampling of chorionic villi from the intrauterine cavity without coming into contact with the vagina, probably infected. We found 16.6% positive cases, therefore, our study undoubtedly demonstrates the presence of HPV in chorionic villi like the studies of Gomez [15] and of Weyn [14], which demonstrated the presence of HPV in placental samples obtained trans-abdominally, while the study of Eppel [13] reports the absence of HPV DNA in 147 placental samples obtained abdominally, probably due to the use of a less sensitive PCR. Furthermore, Chisanga et al. [16] demonstrated the presence of HPV in placental trophoblasts using both polymerase and immunohistochemistry chain reaction methods.

However, the path by which HPV infects the placenta is unknown. It is suspected to occur through the ascending vaginal pathway. In a healthy pregnancy, the growing uterus and fetus are protected from any upward infection by the cervix. The cervix plays a unique role in actively controlling and restricting microbial access through mucus production, inflammatory cytokines and antimicrobial peptides, and therefore represents not only an anatomical barrier but also an immunological barrier against ascending infection recognition receptors such as Toll like (TLR) in the cervical epithelium can detect the presence of microorganisms and elicit an innate immune response characterized by the production of cytokines and AMP [17].

Another possibility could be intrauterine transmission at the time of fertilization by latent HPV-carrying sperm; growing evidence indicates that HPV is found in the sperm of infertile males [18, 19].

The other way is hematogenic as HPV has been detected in the mononucleate cells of peripheral blood, suggesting a hematogenic diffusion path [20, 21]; however, no other data support the spread of the virus through blood. Studies on placental tissues have detected HPV genotypes in aborted tissues, including HPV 6, 11, 16, 18, 58, 66, 82, 83 while a clinical case of verruciform epidermodisplasia also reported the presence of HPV 3, 5, 8, 24, 36 [22]. In our study HPV 16 is the most frequent genotype, while genotype 6 was the second most frequent, in line with data in the literature showing that this genotype is related to vertical transmission. Genotypes 53 and 90 are very rare and little studied, it is thought that they are not related to any oncogenic activity although recently it has been shown that HPV 90 E6 is capable of completely degrading p53 with the same efficacy as HPV 16 E6 in an assay of single transfected cell [23].

The role of papillomavirus in the genesis of cervical cancer is now certain and one important factor is genotyping [24] since genotype 16, which is widespread, is responsible for 70% of cancer cases. During pregnancy, the mutated hormonal environment and immune response may promote the presence or persistence of HPV infection. Data on the prevalence of HPV infection in pregnancy are very discordant: Tenti et al. [25] reported 5.4%, while Cason et al. [26] reported 68.8%. The diversity of observed percentages is related to several factors that alone could influence the results, such as: sample characteristics, inclusion criteria and diagnostic techniques.

The human papillomavirus (HPV), was detected in the cervix in 15–25% of pregnant women [27, 28] and a cervical HPV infection during pregnancy was associated with an increased incidence of miscarriages [29], premature membrane rupture [30], spontaneous preterm birth, pre-eclampsia [31], and placental “villitis” not otherwise specified, however, HPV DNA was also discovered in the placenta of healthy pregnancies [32] and placenta samples obtained trans-abdominally. The relationship between HPV infection and pregnancy outcomes is unclear. Some studies have shown no association, while others have suggested that HPV infection, cervical disease and/or its treatment [33] are associated, in addition to changes in a woman’s sex life [34], with adverse outcomes of pregnancy, such as miscarriage, premature rupture of membranes or preterm birth. Hermonat, showed that HPV type 16 can replicate in trophoblasts [8] and You [5] on cultured 3A trophoblasts cells (chosen as the experimental cell type because they are well characterized and represent a near-normal trophoblast), showed that also other genotypes (11, 18, 31) are capable of reproducing in trophoblast cells. Oncogenic HPVs have pathologically important E6 / E7 bases and their oncogenicity is due in part to their ability to inactivate the cellular tumor suppressor genes, P53 and Rb, respectively. These two oncogenes have a vital functions in the development of HPV-associated cervical cancer. H. You [7] has shown that multiple physiological changes derive from the introduction of E6 and E7 in the trophoblasts 3. In fact, he for to evaluate the effects of E6 and E7, 3A trophoblast cells infected with recombinant adeno-associated viruses, while the AAV / NEO virus infection was used as a control. The investigation shows that there is a lack of recognition of highly defective endometrial cells and therefore a low adhesion of trophoblastic cells to the endometrium, the death of trophoblast cells for E7-induced apoptosis. Knowing the importance of good placentation for a physiological pregnancy, papillomavirus damage to the trophoblast strongly supports an HPV miscarriage link. In summary, the oncogenic component genes of HPV E6 and E7 have significant effects on the general cellular characteristics of trophoblasts, including cell survival, binding to endometrial cells, proliferation, differentiation, and immortalization. One could consider that any of these changes in trophoblasts it could be responsible for the alteration of the trophoblastic and placental physiology and could contribute to spontaneous abortions.

There is clearly a need for further research regarding the relationship between HPV infection and the abnormal course of early pregnancy leading to miscarriage or fetal defects.

While on the one hand our research has a limit represented by the small size of the sample studied, on the other it has a strong point represented by the particular method used to avoid contamination of the placenta with HPV coming from the maternal vagina.

## Conclusion

Our study shows that papillomavirus DNA can infect the placenta and that placenta HPV infection can occur as early as the first trimester of pregnancy. The overall clinical implication of these results remains to be clarified.

## Supplementary Information

**Additional file 1.**

## Data Availability

The dataset used and/or analyzed during the current study are available from the corresponding author on request.

## Abbreviations

STD: Sexually transmitted disease
CIN: Cervical Intraepithelial Neoplasia
HPV: Human Papilloma Virus
TOP: Termination of Pregnancy
