# Supplementary material for: Evidence for HPV DNA in the placenta of women who resorted to elective abortion
Source: BMC Pregnancy Childbirth. 2021 Jul 6;21:485. doi: 10.1186/s12884-021-03937-9 (PMC8258985; doi:10.1186/s12884-021-03937-9)
Supplement: Supplementary file 1 — Additional file 1. [file 12884_2021_3937_MOESM1_ESM.docx]

**Evidence for HPV DNA in the placenta of women** **who resorted to elective abortion**

**Maria Teresa Bruno^1^, Salvatore Caruso^1^, Francesca Bica^1^, Giulia Arcidiacono^1^, Sara Boemi^1^.**

^1^  Department of General Surgery and Medical Surgery Specialties, Gynecological Clinic, University of Catania, Italy

**Author Information**

**Affiliations**

Department of General Surgery and Medical Surgery Specialties, Gynecological Clinic, University of Catania, Italy

Bruno Maria Teresa, Caruso Salvatore, Bica Francesca, Arcidiacono Giulia, Boemi Sara

**Author contributions**

MTB designed the study; GA and SB collected the data; MTB and SC drafted the manuscript; FB compiled the statistical data. All authors were involved in editing the manuscript. All authors read and approved the final manuscript.

**Corresponding Author:** Maria Teresa Bruno

**Supplementary file**

**Tab1. Epidemiological evaluation of study group**

**Age**
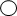
 < 25
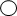
 >25 to <35
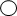
 >35 to 45

**Smoking**
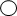
 no
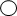
 < 10 cigarettes per day
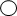
 ≥ 10 cigarettes per day

**Immunodepression**
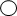
 no
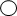
 yes: HIV, …….

**Marital status**
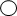
 Married
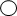
 Single
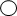
 Cohabiting
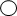
 divorced

**Condom use 
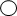
** no
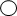
 yes

**Age at first sexual intercorse
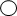
** <15
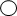
 <20
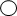
 20-25
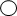
 > 25

**Number of sexual partner
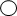
** <2
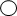
 2-3
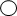
 4-5
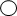
 >5

**HPV infection**
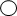
 no
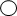
 genital warts
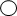
 LSIL
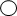
 HSIL

**HPV infection in past year**
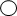
 no
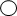
 genital warts
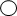
 LSIL
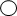
 HSIL

**HPV infection partner**
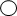
 no
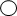
 yes

**Pap test**
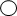
 negative
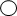
 ASCUS
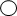
 LSIL
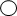
 HSIL

**HPV test**
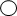
 negative
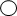
 positive lrHPV
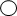
 positive hrHPV

**Gestational age** : _______ weeks
